# A philosophy of health: life as reality, health as a universal value

**DOI:** 10.1057/s41599-020-0420-9

**Published:** 2020-03-18

**Authors:** Julian M. Saad, James O. Prochaska

**Affiliations:** Cancer Prevention Research Center, The University of Rhode Island, 130 Flagg Rd, Kingston, RI 02881 USA

**Keywords:** Medical humanities, Philosophy, Health humanities, Psychology

## Abstract

Emphases on biomarkers (e.g. when making diagnoses) and pharmaceutical/drug methods (e.g. when researching/disseminating population level interventions) in primary care evidence philosophies of health (and healthcare) that reduce health to the biological level. However, with chronic diseases being responsible for the majority of all cause deaths and being strongly linked to health behavior and lifestyle; predominantly biological views are becoming increasingly insufficient when discussing this health crisis. A philosophy that integrates biological, behavioral, and social determinants of health could benefit multidisciplinary discussions of healthy publics. This manuscript introduces a Philosophy of Health by presenting its first five principles of health. The philosophy creates parallels among biological immunity, health behavior change, social change by proposing that two general functions—*precision* and *variation*—impact population health at biological, behavioral, and social levels. This higher-level of abstraction is used to conclude that integrating functions, rather than separated (biological) structures drive healthy publics. A Philosophy of Health provides a framework that can integrate existing theories, models, concepts, and constructs.

## A philosophy of health

What is health? Is it a state of the body or the mind? Is health primarily a natural, biological state or a holistic, value-laden state? Naturalistic and holistic philosophies of health have provided very important, but very different, perspectives of population health. Naturalistic views (e.g. as seen in Boorse, 1997) provide insight into physical, natural, biological, or physiological processes that are tangible (in the material sense), observable, and measurable with modern technology. Complementarily, holistic views contend that value-laden phenomena (e.g. vital goals, meaning, and purpose) play a central role in population health (Nordenfeldt, 2007).

A dialog, or as we see it, an important dialectic among naturalistic and holistic perspectives plays out between the Biostatistical Theory of Health (BST) and the Holistic Theory of Health (HTH). The BST posits that a person is healthy if and only if, *all natural organs function normally* given a statistically normal environment (Boorse, 1997). The HTH posits that a person is healthy if and only if (given standard circumstances) he/she has the *ability to attain their vital goals* (Nordenfeldt, 2007).

In addition to defining health, each philosophy defines disease. The BST poses that disease is the internal state of impairment to the normal functioning of organs (Boorse, 1997). In the HTH, an organ dysfunction is a *disease* if and only if the organ’s process reduces the person’s ability to pursue vital goals or life-purpose (Nordenfelt, 2007). In BST health *is* the absence of disease; and in HTH, health *is not* the absence of biological disease, but is the whole person’s ability to function in relation to vital goals.

Both naturalistic and holistic perspectives guide important observations of health and disease. When one considers health through the BST one pays close attention to the functions of the internal, biological functioning of the human being. When one considers health through the HTH, one pays close attention to the functioning of an individual, in relation to their external, societal/cultural functions. Is there a hybrid model that accounts for both internal and external functioning?

Wakefield’s (2014) harmful dysfunction analysis (HDA) creates a hybrid model that integrates natural- and value-laden phenomena when conceptualizing disease. HDA asserts that a person suffers from a disorder/disease if (1) the condition causes *harm* (as judged by the standards of the person’s culture); or if (2) the person’s internal, natural processes cannot perform *normal functioning* (as judged by the standards set by evolution). HDA creates a hybrid model that can integrate perspectives of the BST (i.e. by considering internal organ functioning); and the HTH (i.e. by considering external societal/goal functioning). However, while HDA may define health processes in relation to disease, it serves primarily as an integrative model of *disease*. Is there an integrative model of *health* that can account for natural and value-laden functions?

Schroeder (2012) identifies a significant, common thread among these competing (or perhaps complementing) philosophies: *functionalism*. The researcher suggests that each philosophy is concerned with the functioning of organisms. Although the BST, HTH, and the HDA might not agree on which functions inform the first principles of health, Schroder (2012) uses higher-level abstraction to identify one common first principle: the state of *functioning* in an organism impacts its state of *health*. When paralleling the three philosophies based upon functioning one might observe that (1) BST declares an individual healthy if its organs function normally; (2) HTH declares an individual healthy if he/she can function in relation to vital goals; and (3) HDA declares an individual unhealthy if internal mechanisms cannot perform natural, evolutionary functions, and/or when a condition prevents a person from functioning in relation to goals/norms/values. Through this higher-level abstraction, an integration of seemingly separate philosophies of health is made possible.

### Learning from leaders in the field

As we attend to these philosophies of health, we too observe how discussions about functions and functioning produce integrative perspectives. Although a definition of “function” is not explicitly stated in the above research, it appears that Nordenfeldt (2007), Boorse (1997), Wakefield (2014), and Schroeder (2012) are each discussing functions as pre-existent (i.e. either from evolution, personal goal-setting, cultural tradition) *processes*-*with*-*purposes*. Whether one is describing a value-laden function (e.g. decision-making in pursuit of a valuable career) or an evolutionary-biological function (e.g. the heart beating for circulation), each process (i.e. decision-making processes or cardiac processes) serves identifiable purposes (e.g. maintained financial stability or maintained blood flow). Whether an organ is functioning normally in relation to the body or a human being is functioning in relation to vital goals, it appears that both perspectives consider if an active “process” (i.e. an organ’s activity, an individual’s activity) can express its “purpose” (i.e. evolutionary-purpose, life-purpose).

In the present manuscript we will propose that naturalistic and holistic perspectives can be integrated within a single philosophy of health. We will propose two universal functions—termed *precision* and *variation*—that can account for both natural functions and value-laden functions of the existing philosophies. This functional language will support a higher level of abstraction that integrates, rather than separates, biological functions, behavioral functions, and social functions under A Philosophy of Health.

### The need for new perspectives in population health

The chronic disease crisis beckons the need for an updated philosophy of health that can account for biological, behavioral, and social functioning. Why? Chronic diseases, which account for 60% of all-cause deaths worldwide (Chartier and Cawthorpe, 2016), do not emerge from naturalistic, biological, or physical contact with an illness. Rather, chronic diseases do emerge in biological functions (e.g. tumor proliferation in an organ) after prolonged contact with health risk behaviors and lifestyle factors that active the conditions (Mokdad et al., 2018; Edington, 2009; Li et al., 2018). Chronic diseases are not curable by purely naturalistic or biological means (e.g. pharmaceuticals). Rather, some diseases may be effectively prevented or intervened on through healthy behavior (Dansinger et al., 2005; Daubenmier et al., 2007).

Population health risk behaviors are unique determinants of population health because researchers can actively observe how they simultaneously alter biological functioning (e.g. chronic smoking alters cells in lung tissue), behavioral functioning (e.g. chronic smoking alters decision-making and daily habits) and social functioning (e.g. chronic smoking creates an economic, social, and healthcare burden) of the population. These behaviors not only have biological, behavioral, and social implications for the individual *doing* the behavior, but also have intergenerational and interpersonal effects. The individual who binges on refined sugar not only puts themselves at risk of diabetes, but can put their future offspring at risk. The individual who smokes two packs of cigarettes per day not only puts themselves at risk of lung cancer, but can put their housemates at risk of lung cancer from second-hand smoke. Therefore, the chronic disease crisis is neither purely naturalistic, nor purely value-laden; rather it reflects an integration of natural and value-laden phenomena. There remains a real need for principles of health that can integrate existing naturalistic and holistic perspectives of population health.

## The principles

Since April 7, 1948, the Constitution of the World Health Organization (2010) has utilized an intuitive definition of health by suggesting that health is “a state of complete physical, mental, and social well-being.” While this definition might be intuitive and even accessible to a wide audience; the defininition *is not* necessarily researchable across health disciplines. Integrating principles of health might begin with a common-sense definition of health that can also be upheld across existing naturalistic and holistic perspectives. Without operationally defining functions that drive physical, mental, and social well-being, it is a challenge for multidisciplinary collaborators to unite under the WHO mission. Further, without a common definition of health, important communications from patients to doctors, from subjects to researchers, from researchers to collaborators, and from peer-reviewers to peer-reviewees, can become fragmented or lost in translation. In the proceeding sections, a common-sense definition of health is used to present the first principles of A Philosophy of Health.

### Principle 1: “Health” is the state of *maintainable-easeof functioning*. A “disease” is a state of *prolonged-dysfunction that prevents ease*

Chronic diseases emerge from prolonged exposure to dysfunctional behaviors like smoking, alcohol abuse, unhealthy diet, and inactivity (Mokdad et al., 2018) that also create dysfunctional expressions of life functions. Smoking creates dysfunctional breathing; alcohol abuse creates dysfunctional drinking; sugar binging creates dysfunctional eating; and sedentary behavior creates dysfunctional moving. When these health risk behaviors lead to chronic disease, they have already *prolonged dysfunctional* breathing, drinking, eating, and/or moving.

The chronic smoker breathes in smoke so frequently that he no longer experiences an ease-of-breathing. Rather, his breathing becomes short and shallow. Prior to the emergence of lung tumors, the chronic smoker prolongs dysfunctional patterns of breathing. The “couch potato” sits so frequently that he no longer experiences an ease-of-movement. Rather his movement becomes rigid and limited. Prior to the emergence of cardiovascular dysfunction or obesity, the sedentary person prolongs dysfunctional patterns of movement.

If chronic smoking facilitates prolonged-dysfunction in breathing, and sedentary behavior facilitates prolonged-dysfunction in movement, what do functional breathing and moving look like? Healthy breathing and moving (as well as eating and drinking) are characteristic of an *ease* of one’s *functioning* that can be *maintained* in normal conditions. For example, the chronic smoker and the “couch potato” might report momentary-ease in breathing and posture when engaging in their health risk behaviors; but they *do not maintain* that ease outside of smoking or sitting. Conversely, the yogi might report that their yoga practices expose them to momentary dis-ease in breathing and moving that lead to maintainable-ease in breathing and movement in everyday life. In contrast to disease as a prolonged-dysfunction, healthy functioning can be commonly sensed as a *maintainable*-*ease of functioning*.

When observing a disease, perhaps we are observing a *prolonged-dysfunction* that prevents ease. Rather than define health as the absence of disease (as seen in BST), notice here how we instead define disease in relation to health; and we define health in relation to *maintainability*, *ease*, and *functioning*. Consideration of “maintainable-ease of functioning” will allow us to consider how not all “dis-ease” is bad (i.e. exposure to acute dis-ease/stress maintains healthy functioning in the long-term); and not all “ease” is good (i.e. avoidance of stress and prolonged “comfort” creates fragility seen in sedentary behavior). We propose that:

1. Dysfunction parallels a state of “dis-ease”; and *prolonged*-dysfunction parallels the state of Disease.
2. Function parallels a state of “ease”; and maintainable-ease of functioning parallels the state of Health.

This definition of health will be applied in the proceeding principles to integrate naturalistic and holistic perspectives of population health.

### Principle 2: Health emerges from maintainable-ease of functioning at multiple *levels*. Maintainable-ease of functioning in the general population can be observed at the level of the *cell*, the *self*, and the *society* simultaneously

Cooperation across multiple levels of functioning is required for the organization and adaptation of living systems (Nowak and Sigmund, 2005; Antonucci and Webster, 2014). When developing an integrative model of health, it is important to consider how biological cells, individuals, and the larger society simultaneously play a role in population health (Xavier da Silveira dos Santos and Liberali, 2019; Antonucci and Webster, 2014). In this philosophy, we define health from three levels: *cells*, *selves,* and *societies*. What happens when these levels do not function in cooperation?

When the functioning of cells disrupts the functioning of the self, a state dis-ease in the self can follow. For example, prolonged dysfunction in autoimmune conditions can lead to prolonged dysfunction for the (individual’s sense of) self by triggering depression, decreased motivation, or anxiety (Lougee et al., 2000; Garud et al., 2009). The reverse can also be true. When the functioning of the self (i.e. one individual) disrupts the functioning of their cells, a state dis-ease in the cells can also follow. For example, prolonged sugar binging and addictive eating can lead to prolonged high blood sugar and pancreatic dysfunction seen in diabetes (De Koning et al., 2011; Imamura et al., 2015). Cells and selves are not separate.

When the functioning of the self disrupts the functioning of the society we observe a state dis-ease in the society. For example, one person’s unprotected sex with multiple partners can also lead to epidemics and social conflicts. The reverse can also be true. When the functioning of the society disrupts the functioning of the individual, a state dis-ease in the self can follow. For example, dysfunctional social conditions (as seen in Rutter, 1998), can lead to prolonged psychological and behavioral dysfunctions of individuals. Selves and societies are not separate.

When the functioning of society disrupts the functioning of cells, a state of dis-ease in the cells can also follow. For example, prolonged dysfunction in society in the form of misguided values about cleanliness, can lead to over-sanitization practices that create superbugs and antibiotic-resistant bacteria (Zaccheo et al., 2017; Finkelstein et al., 2014; Bower and Daeschel, 1999). The reverse can also be true. When the functioning of cells disrupts the functioning of the society, a state of dis-ease in the society can follow. Prolonged dysfunction in cells from naturally occurring parasites (e.g. *Yersinia pestis* [Cui et al., 2013]) can lead to prolonged dysfunctions like the economic collapse following 14th century Black Death (Haensch et al., 2010). Cells and societies are not separate.

What does health look like when these levels work together? Recent reports on the Blue Zones (i.e. the areas of the world where populations live significantly longer and healthier than the average) demonstrate that healthy functioning at these levels enhances physical longevity and mental wellbeing in populations (Buettner, 2012; Poulain et al., 2013). Buettner (2012) reports on how Blue-Zone populations intentionally and habitually enrich their physical bodies with healthy eating and physical activity. In addition to integrating physical and behavioral practices, these communities also integrate behavioral and social practices, such as, goal-setting, meditations/prayer, social engagement, pursuit of purpose, and community gathering. Humor is used by individuals and groups as a means to practice ease when challenges present themselves (Buettner, 2012). Blue Zone communities place value upon physical/natural, behavioral and social processes, generating them intentionally and habitually.

**Fig. 1:**
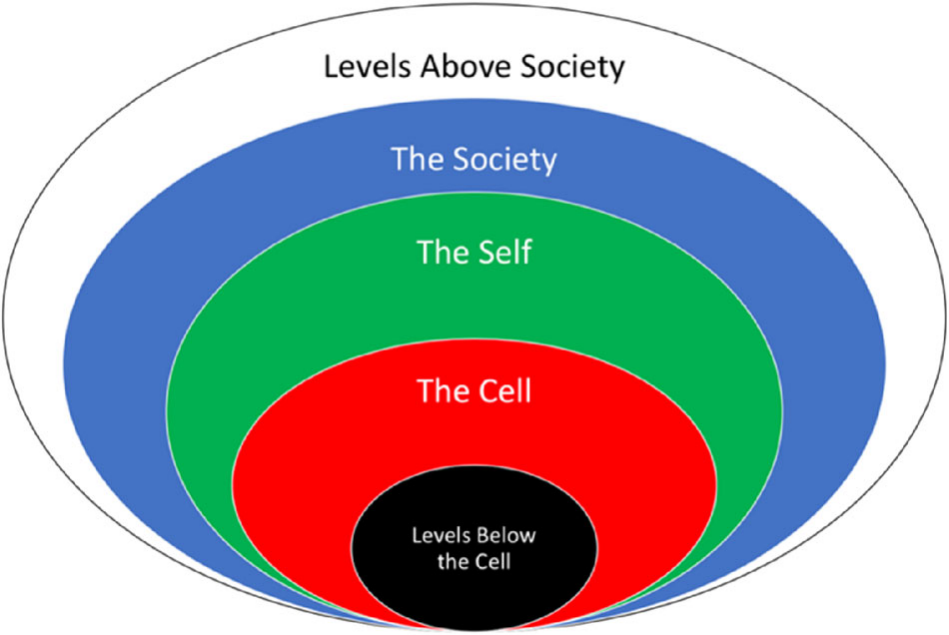
The levels of functioning. This philosophy of health investigates three levels of health: cell, self, and society. The level of the cell accounts for biological functioning within human beings. The level of the self accounts for first-person functioning of each human being. The level of the society accounts for group functioning of human beings.

Both states of ease and dis-ease can teach us about the contributions of cells, selves, and societies to population health. Although it is important to be able to observe the levels separately to describe their contributions, it is also important to consider how the levels integrate to impact healthy publics. We acknowledge that meaningful changes can be observed above and below these levels (e.g. at the level of the biosphere and genome). However, this initial paper will introduce levels that are most proximal and accessible to the experience of a general readership (Fig. 1).

### Principle 3: Health emerges from *systems* whose primary purpose is to generate *maintainable-ease of functioning* at a respective *level*

**Fig. 2:**
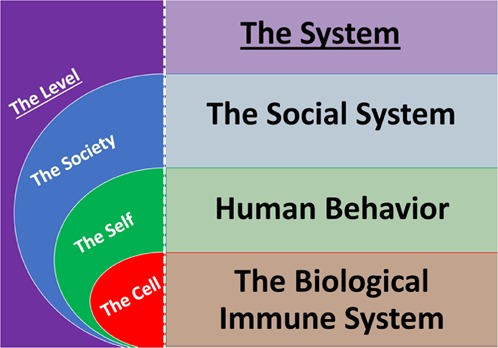
The systems at each level. Each system is responsible for generating maintainable-ease of functioning at a level. The biological immune system is responsible at the level of the cell. A human's system of health behaviors is responsible at the level of the self. The social system is responsible at the level of the society.

We propose that *systems* exist at each level with the purpose of generating maintainable-ease of functioning at that level. The biological immune system, an individual’s system of health behaviors, and the social system will be observed as *systems* that generate *maintainable-ease* of functioning in cells, selves, and societies respectively (Fig. 2).

#### Principle 3a: *The biological immune system* is directly responsible for maintainable-ease of functioning at the *level of the cell*

Throughout the course of human evolution, the complexity and biodiversity of the human body continued to increase (Rodríguez et al., 2012). What keeps the trillions of cells and microorganisms in cooperation in a human body? The biological immune system maintains functional cells (Rodríguez et al., 2012). Although it is documented that the functioning of the biological immune system has implications for behavioral functioning (Ader, 1974, 2000; Johnston et al., 1992; CDC, 2016) and social functioning (CDC, 2016; Reidel, 2005; Cutler and Miller, 2005) the system’s primary *purpose* is supporting functioning in the cellular/biological system.

#### Principle 3b: *Health behavior* is directly responsible for *maintainable-ease* of functioning at the *level of the self*

Throughout the course of time, the complexity of human behavior, has continued to increase (Boulding and Khalil, 2002). What keeps an individual in a state of balance during times of rapid change? One’s system of health behaviors (e.g. one’s practices of breathing, drinking, eating, and moving) maintain a functional self. Although it is well documented that the behavior of the individual impacts biological functioning (Fadel, 2013, 2015) and social functioning (Omer et al., 2009), one’s system of health behaviors directly impacts one’s experience of (or one’s ‘sense of’) their “self”.

#### Principle 3c: *The social system* is directly responsible for *maintainable-ease* of functioning at the *level of the society*

Throughout history, the social diversity of human societies continued to increase. During periods of rapid increases in social diversity and cultural integration, what supported cooperation in the society? Social systems (e.g. public governments, private social organizations, religious/spiritual organizations) emerge to maintain a functional society. Although it is well documented that a social system can impact biological functioning (CDC, 2016; Riedel, 2005; Cutler and Miller, 2005) and behavioral functioning (Buettner, 2012), the social system’s primary role is to maintain functions at the level of the society.

#### Principle 3d: By considering health as *maintainable-ease of functioning* generated by *systems*, we have the ability generalize health across *levels*

To observe health at the level of the cell, the self, and the society simultaneously, we consider systems that support maintainable-ease of biological, behavioral, and social functioning. The biological immune system, an individual’s system of health behaviors, and the social system make meaningful contributions to the functioning of cells, selves, and societies, respectively. While these systems are not the only systems that impact each level (e.g. one’s cardiovascular system impacts cells, one’s “personality” impacts the self, the environment impacts society), the biological immune system, health behavior, and the social system have great implications for population health from their respective levels; *and* they can be operationalized at these levels based upon their *functions*.

By considering health as maintainable-ease of functioning (rather than maintained biological structures) at multiple levels, we set a point of reference from which to integrate important determinants of population health. When taking the *structuralist’s* perspective, the biological immune system, health behavior, and social systems appear as distinctly separated. When taking a *functionalist’s* perspective, the biological immune system (i.e. the integration of host defense functions and microbiota functions), one’s (system of) health behaviors (i.e. the integration of decision-making/executive functions and habits/habitual life functions), and the social system (i.e. the integration of population values and population behaviors) appear together in A Philosophy of Health.

### Principle 4: Each system employs two general functions—*variation* and *precision*—to generate maintainable-ease of functioning at a level

The functionalist perspective allows us to observe systems based upon their *functions*. The biological immune system will be observed as an integration of *host defense functions* and *microbiota functions* (Hooper and Littman Macpherson, 2012); (2) an individual’s system of health behaviors will be observed as an integration of *decisions/executive functions* and *habits/habitual life functions* (de Bruin et al., 2016; Verplankern, 2005; Norman et al., 1998; Prochaska et al., 1994; Prochaska et al., 1991); and the social system will be observed as an integration of actively functioning *values* and *population-wide behaviors* that function in relation to those values (Dowling and Pfeffer, 1975; Cotgrove and Duff, 1981).

By researching the role of these functions at each level, we distilled two general functions of each system: variation and precision. *Variation* appears in the functions of each system that generate a range of abilities, the “varied-abilities”, that sustain health in presently changing conditions. The microbiota, habits/habitual life functions and population behaviors will be observed (in Principle 4a) as the variation-functions of the biological immune system, health behavior, and the social system, respectively. *Precision* appears in those functions that prioritize and organize the patterns of variation that can sustain health at a level in future, changing conditions. The host-defense functions, decision-making/executive functions, and values systems will be observed (in Principle 4b) as the precision-functions in the biological immune system, health behavior, and the social system, respectively.

**Fig. 3:**
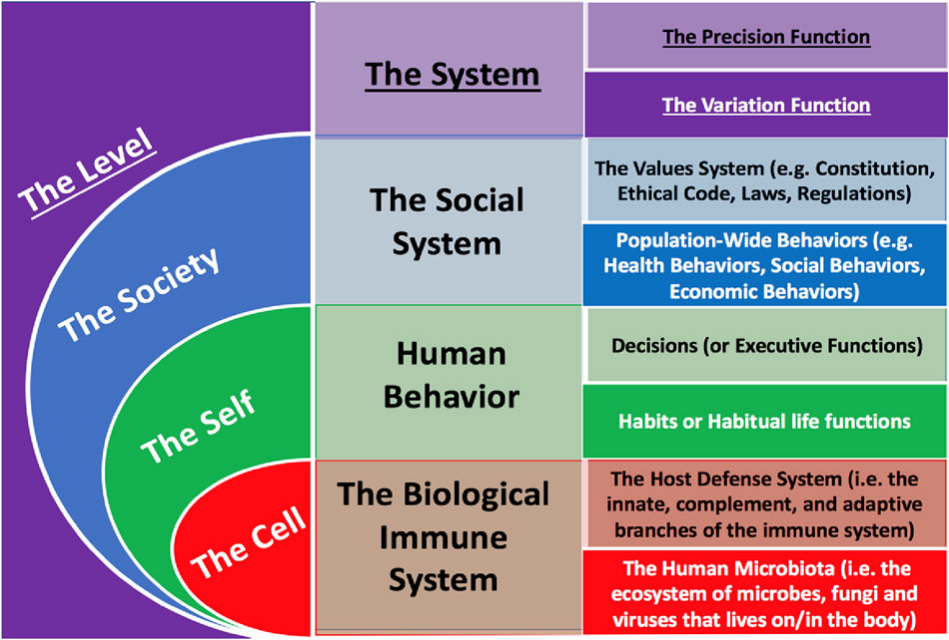
Precision and variation in each system. Maintainable-ease of functioning is generated by two functions in each system: precision and variation. The human microbiota, habits, and population-wide behaviors evidence variation in cells, selves and societies respectively. The host defense system, decisions, and values evidence precision in cells, selves and societies respectively.

Consideration of a complementary relationship among precision and variation is not novel. Precision and variation have been discussed as central to the development of neural and biological systems (Hiesinger and Bassem, 2018). Discussions of precision and variation have also provided important insight into research on the biological immune system (Albert-Vega et al., 2018; Brodin et al., 2015). Through this philosophy, one can go beyond biological systems to observe how precision (in the form of host-defense functions, decision-making/executive functions, and values) and variation (in the form of microbiota functions, habits/habitual life functions, and population-wide behaviors) integrate to generate to maintainable-ease of functioning in cells, selves, and societies simultaneously (Fig. 3).

#### Principle 4a: *Variation* is responsible for generating the range of abilities, the “varied-abilities”, that can express ease-of-functioning in presently changing conditions

Without functional variation, life is fragile because the present environment is always changing (Taleb and Blyth, 2011). Fragile systems’ inability to experience changing conditions (in part) relates to limited variability. Conversely, adaptive system’s ability to experience changing conditions (in part) relates to functional variability (Taleb, 2012). When one microorganism in the microbiome takes over, biological fragility reflects a state of infection. When one habit takes over, behavioral fragility reflects a state of an addiction/dependence. When one population behavior takes over (e.g. when economic participation or access to food is restricted to a small percentage of the population) social fragility reflects a state of social/civil unrest.

The human microbiota is comprised of trillions of microorganisms, such as bacteria, fungi, and viruses. When variability in the human microbiota exists, an ease of functioning, or “homeostasis” in cells can be expressed in the present biological/ecological environment (Parfrey and Knight, 2012; Bogaert et al., 2011; Claesson et al., 2011). Research demonstrates that *variation* in the microbiota impacts the health of human cells by metabolizing complex carbohydrates, converting proteins to neural signals, and modulating diurnal rhythms that maintain biological homeostasis (Clemente et al., 2012; Rothe and Blaut, 2012; Blaut and Clavel, 2007; De Vadder et al., 2014). When variation in the microbiota is dramatically limited or changed (e.g. following antibiotic overuse), cellular tissue in the human body is fragile and vulnerable to infections, allergies, and inflammatory outbreaks (Francino, 2016).

When one’s habitual life functions (e.g. breathing, drinking, eating, and moving) and one’s healthy habits (e.g. one’s weekly exercise schedule, or weekly meal preparation) can be expressed freely, an ease of functioning is felt by one-self in the present environment. When life functions are no longer expressed with ease (e.g. breathing and movement are compromised due to prolonged sedentary lifestyle), or when a single habit takes over one’s lifestyle (e.g. smokes breaks “must” occur every 30 min), an individual is vulnerable to stressful outbreaks and chronic states (Al’Absi, 2011; Conrad et al., 2007; Suess et al., 1980; León and Sheen, 2003; Parrott, 1999; Koob, 2008).

When the basic human rights in a society are preserved in the present (e.g. right to life, freedom of speech; right to property), human populations have the ability to freely engage in the *population*-*wide behaviors* (e.g. health behaviors, social behaviors, economic behaviors) that support a functioning society. Health behaviors drive health and longevity. Social behaviors drive communication and cooperation. Economic behaviors drive goods and resources. When these population-wide behaviors are chronically restricted in a population (e.g. poor access to health care, oppression of free-speech, economic crash), societies become vulnerable to social/civil unrest [as commented historically by Victor Frankl (1985), Alexander Solzhenitsyn (2003), Franklin D. Roosevelt (1941), and Dr. Martin Luther King (1985)].

Variation is essential so that a system has varied-abilities that can *express ease-of-functioning in present environmental conditions*. Dramatic and prolonged restrictions to variation in the microbiota, habits/habitual life functions, and population-wide behaviors characterize fragile and vulnerable states in cells, selves, and societies. Conversely, functional-variation supports resilience, robustness, and antifragility (Taleb, 2012). This does not mean that infinite variation is desirable; however, in this philosophy, *precision* is responsible for organizing expressions of variation so that the system does not degrade into unpredictably random variation or chaos (see Principle 4b).

#### Principle 4b: *Precision* is responsible for prioritizing and organizing the patterns of variation that maintain ease-of-functioning in future, changing conditions

Some environmental changes are too challenging for ease to be expressed in the present. However, following an exposure to challenging conditions, some systems adapt and become more functional (Taleb, 2012). Without the ability to functionally organize after stressors, a system degrades into disorder or chaos over time. Host-defense functions, decision-making/executive functions and values systems prioritize and organize variation in the microbiota, habits/habitual life functions, and population behaviors respectively.

When a pathogen invades the biological system, precise responses must occur to organize this potentially chaotic situation. At the level of the cell, a functional host-defense system (comprised of the innate, adaptive and complement immune system branches) organizes the biological system so that functional invaders (i.e. symbionts) and healthy cells are maintained and dysfunctional invaders (i.e. pathogens) and damaged cells are removed (Hoeb et al., 2004; Janeway, 1992; Janeway and Medzhitov, 2002; Janeway et al., 2014). When precision is dysfunctional, the host-defense system may (1) fail to prioritize responses to a costly invasion, leading to a state of infection; or (2) the host-defense system might prioritize dysfunctional responses to the cells of body that prolong a state of autoimmunity (Naor and Tarcic, 1982).

When a bad habit emerges, precise responses must occur to organize this potentially chaotic situation. At the level of the self, functional decision-making (or at smaller scales executive functioning) prioritizes and organizes behavior so that functional expressions of habit (or at smaller scales, habitual life functions) are prioritized regularly, and dysfunctional ones are replaced or minimized (de Bruin et al., 2016; Prochaska et al., 1994; Prochaska and Prochaska, 2016; Prochaska et al., 1988; Redding et al., 2011; Weissenborn and Duka, 2003; Bickel et al., 2012). When dysfunctional, decisions may (1) fail to prioritize responses that remove a costly expression of habit (e.g. a teen started smoking cigarettes to “be cool” and now has to smoke in the bathroom before each class to get through the day; by not deciding to move at work, one’s breathing becomes shallow and movement becomes rigid); or decisions may (2) prioritize habits that prolong dysfunction despite knowing the dangerous consequences (e.g. an adult continues smoking cigarettes despite knowing the family’s history of lung cancer; an adolescent continues binge on sugar despite a diabetes diagnosis).

When dangerous population-wide behaviors threaten life in a society, precise responses must occur to organize this potentially chaotic situation. At the level of society, the agreed upon values organize the social system so that functional population behaviors are prioritized and dysfunctional population behaviors are minimized. Functional values prioritize behaviors that support the society (e.g. as seen when societies mandate that students get certain vaccines before attending University), while also setting standards that remove/replace behaviors that threaten the society (e.g. new laws create legal repercussions for risk behaviors in society). Without values that functionally prioritize population-wide behavior, society may (1) fail to prioritize responses to a dysfunctional population behavior (e.g. as seen during AIDS epidemic of the 1980s due to insufficient public health values around safe sex); or society may (2) prioritize dangerous behaviors that can prolong societal dysfunction (e.g. the antibiotic resistance crisis (Ventola, 2015; Michael et al., 2014) has been attributed in part to the over-valuing or over-use of antibiotic medications in healthcare practices).

Precision is essential so that a system can *maintain ease-of-functioning in future, changing conditions*. When precision does not adequately detect the presence of costly conditions, a response may not be prioritized (e.g. as seen during acute infection, addiction/dependence following a surgery, the AIDs outbreak in the 1980s). When precision prioritizes responses that prevent ease longitudinally, dysfunction is prolonged (e.g. autoimmunity, continued smoking despite family history of cancer, misguided values that create an antibiotic-resistant bacteria). Through *dysfunctional*-precision, the conditions for life in cells, selves, and societies becomes disordered over time. Through *functional*-precision, a system prioritizes responses that maintain ease-of-functioning in future conditions. Prioritizing functional microorganisms (i.e. symbionts) supports the developing life of cells; prioritizing functional habits (e.g. weekly exercise) and habitual life functions (e.g. diaphragmatic breathing and relaxed movement) supports the developing life of the self; and prioritizing functional population behaviors (e.g. access to functional health care, economic resources; access to social support) supports the developing life of the society.

### Principle 5: Health is *valued* by a system when precision-and-variation generate maintainable-ease of functioning. Health is *de-valued* by a system when precision *or* variation prevent maintainable-ease of functioning

By defining precision-and-variation, we can better understand maintainable-ease of functioning in population health:

- Functional-Variation *generates ease-of-functioning in the present* (e.g. fluid and variable motion reflects an ease and variability of one’s movement); while Functional-Precision prioritizes expressions that can *maintain ease-of-functioning in the future* (e.g. prioritizing challenging exercise for 20 min each day may lead to an ease in bodily movement long term).
- Dysfunctional-Variation *prevents ease-of-functioning in the present* (e.g. prolonged sitting might lead to rigid movement and shallow breathing); while Dysfunctional-Precision might prioritize expressions that *prevent ease in the future* (e.g. rather than focus on relaxing breathing and movement on work breaks, one decides to drink alcohol to relax).

Without functional-variation, life is fragile and vulnerable to changing conditions of the present. Without functional-precision, life becomes disorganized from the system’s exposure to changing conditions across time. When functional-and-integrated, precision-and-variation *value* maintainable-ease of functioning in cells, selves, and societies. When dysfunctional or fragmented, precision or variation can *de-value* maintainable-ease of functioning in cells, selves, or societies. If maintainable-ease of functioning can be valued in cells, selves, and societies, we will likely observe healthy publics.

## Discussion

Five principles of health are presented: (1) Health is the *maintainable-ease of functioning;* (2) Maintainable-ease of functioning emerges from multiple *levels*; (3) At each level, maintainable-ease of functioning is generated by *systems*; (4) Each system employs two functions, *precision*-*and*-*variation*, that generate maintainable-ease of functioning*;* and (5) Health is *valued* by a system if precision-and-variation generate maintainable-ease of functioning. Through these five principles, both naturalistic and holistic perspectives can be considered simultaneously because maintainable-ease of functioning is relevant to biological functioning (e.g. as described in BST) and personal/social, goal-oriented functioning (e.g. as described in HTH). This philosophy can also be used to investigate how naturalistic and holistic phenomena have informed past healthcare interventions. What do vaccine interventions, behavior change interventions, and social change interventions have in common? When successful, these interventions enhance both precision and variation.

Vaccine interventions can enhance both the *precision* of the host-defense functions and *variation* in the microbiome. During a vaccine intervention, the microbiome is exposed to a new variation in the form of a new virus (Reidel, 2005). Through this exposure, the precision of host defense functions can adapt to prioritize maintainable-ease of functioning in the microbiome in the future. How? The host-defense system produces antibodies that allow the immune system to respond effectively and efficiently to this virus when exposed to it again in the future (Janeyway, 2014). Although the precision of the immune system has been enhanced to handle historical threats through vaccines (e.g. for small pox, chickenpox, measles), new viruses like the coronavirus can still emerge. With this philosophy, vaccine developers and public health officials might not only ask the question, “How do we combat the coronavirus?” Researchers, vaccine developers and public health officials may also ask the functional question: “How do we enhance the precision of the host-defense system and the variation of the human microbiome to adapt following an exposure to the coronavirus?”

Behavior change interventions can enhance both the *precision* in one’s decisions and the *variation* in one’s habits. During a behavior change intervention, a person’s existing habits are exposed to a new variation in habit. For example, the beginning of a new exercise intervention exposes the individual’s current habits/habitual functioning to changes in movement and breathing (i.e. exercise) that may also change their patterns of eating and hydration. Through this exposure, a person’s decision-making might adapt to prioritize maintainable-ease of functioning in the individual’s lifestyle. How? Some behavior change interventions train one’s decision-making to remove or “counter-condition” unhealthy habits, by replacing them with healthy habits (Prochaska et al., 1988). Although modern behavior change interventions have shaped the precision of decision-making during health behavior change (e.g. of smoking, diet, alcohol use, inactivity), new problems for health behavior still emerge when the individual is exposed to a new, potentially addictive technology. With this philosophy, behavior change interventionists and health officials might not only ask the question, “How do we support good decision-making of individuals?” Researchers, behavior change technology developers, and public health officials may also ask the functional question: “How do we enhance the precision of one’s decisions and the variation of one’s habits following the exposure to a new, potentially addictive technology?”

Public health campaigns disseminated by social organizations can enhance the *precision* of the population’s health values and *variation* in population-wide health behaviors. Leading up to first Surgeon General’s Advisory Committee on Smoking and Health (1964), the U.S. Department of Health had become increasingly aware of (i.e. exposed to) variations in a population health behavior. If populations smoked, then populations were more likely to develop lung cancer, laryngeal cancer, or chronic bronchitis (CDC, 2018). Following this exposure to (the consequences of) population smoking behavior, society’s values shifted to prioritize health. How? The Federal Cigarette Labeling and Advertising Act of 1965 was adopted, and the Public Health Cigarette Smoking Act of 1969 was adopted to create new health values. This shift in values prioritized new variations in population health behavior by: (1) requiring a health warning on cigarette packages; (2) banning cigarette advertising in the broadcasting media; and (3) calling for an annual report on the health consequences of smoking (CDC, 2018). Since these first initiatives adult smoking rates have fallen from about 43% (in 1965) to about 18% today; and mortality rates from lung cancer, the leading cause of cancer death, are declining (Department of Health and Human Services, 2014). Although the precision of the population’s values has been enhanced to impact population behaviors (e.g. the tobacco laws described above supported healthy change), new chronic states can still emerge following exposure to social changes (e.g. the invention of the Juul impacted high school and college aged populations). With this philosophy, public policy officials and public health researchers might not only ask the question, “How do we create new laws to protect population health from nicotine addiction?” They may also ask the functional question: “How do we enhance the precision of the population’s values and the variation of the population’s behavior following the invention of a new nicotine delivery system technology (e.g. flavored Juuls)?”

Previously we described that without functional variation, life is fragile when exposed to present changing conditions; and without functional precision, life becomes disorganized from exposure to changing conditions across time. When successful, the above interventions upon biological, behavioral, and social functioning have a common theme: each facilitates *exposures* to biological, behavioral or social conditions that support (1) increasingly complex/diverse variation; and (2) increasingly organizable precision. Exposure, *not avoidance*, has facilitated population health in these interventions. While healthcare systematically prioritizes biological exposures in the form of vaccine interventions, they *do not* systematically prioritize behavioral or social exposures. However, it is documented that exposure to healthy behaviors in youth prevents risk behaviors in adolescence (Velicer et al., 2000); and exposure to community-based health initiatives can support population health (Dulin et al., 2018; CDC, 2018). Given that systematic biological exposures in the form of vaccination have led to a global control of some acute infectious diseases (Tangermann et al., 2007); might systematic behavioral and social exposures (especially in youth) be needed to enhance global campaigns toward the control of chronic disease?

A functional language of health is central to the success of a Philosophy of Health. Why? The levels are not separate, but rather are continuously connecting with one another. A good philosophy of health should have the ability to discuss assessment, diagnosis, intervention, *and* prevention across levels, across systems, across cultural populations, and across time. Using the common language of precision and variation creates discussions that connect the levels and integrate research disciplines.

### A case (to) study: mental health as between-level functioning in this philosophy

Historically, and still too often, health professionals have an expertise at one level, that limits their prescription of interventions to that level. This can actually create barriers to a complete solution when a health problem is multileveled. While a person’s mental health is typically assessed based upon their first-person experience of thoughts, feelings, and behaviors; symptoms can be triggered by biological, physiological, behavioral, psychological, and/or social dysfunction. Most clinicians typically do not have the ability to assess and address all forms functioning. So if one person, John, is meeting with a clinician who specializes in primary care medicine, he may only be prescribed a biological intervention like medication. If John is meeting with a clinician who specializes in behavioral medicine, he may only be prescribed a health behavior change intervention. If John is meeting with a clinician who specializes in a certain theory of psychotherapy, he may only be prescribed a psychotherapy intervention based on the clinician’s training. If John is meeting with a clinician who specializes in social work, he may only be prescribed a group, community or social intervention. While the above specializations have been helpful in establishing an empirical bases for mental health interventions, over-specialization can be problematic when a multi-leveled solution is needed. In addition, it can also be problematic when a level-specific solution is needed that the clinician cannot provide (e.g. when psychotherapy is needed but a clinician only has the ability to prescribe psychiatric medication).

Technology poses a multileveled issue for population mental health in 2020. Selves have more social connection then ever in history, yet societies are characterized by increasing rates of depression and loneliness (Sum et al., 2008; Hammond, 2020; Srivastava and Tiwari, 2013; Twenge, 2017). Researchers might use this Philosophy of Health to facilitate between-level conversations that address seemingly paradoxical outcomes that emerge during this new age of rapid technological growth. To do this, a researcher might *first* begin by asking questions about functioning *at each level*; *second,* ask questions about processes *between the levels*; and *third,* concurrently ask questions *at and between levels*.

#### First: Begin by asking questions at each level

Novel challenges face the iGeneration (and their parents) due to technology’s novel impacts on the development of individual and social functioning (Twenge, 2017). For example, if John’s decisions (self-precision) and habits (self-variation) remain consistent during school hours because his parents do not let him have a phone; but his class’ social behaviors around him (society-variation) change dramatically because everyone else at school uses the newest smartphone application to talk during class; will John’s mental health suffer? Although his parents’ intentions are to protect John, the contrast between his behavior (self’s precision-and-variation) and the population social behavior (society-variation) can impact John’s health. Notice here how we have not yet considered functions that connect the self to the society (e.g. John’s thoughts and feelings). Rather we first consider (or contrast) functioning *at the level* of the self (i.e. John’s decisions-and-habits) and the society (i.e. population social behavior) in accordance with Principles 1–5 (see Figs 1–3).

#### Second: Look for functional processes that connect the levels

One person’s thoughts and emotions/feelings are processes that help to *integrate* the functioning of one-self within the functioning of a society. How might John’s thoughts and feelings connect his (sense of) self to his society? Perhaps John’s parents teach him that it is important to *feel* separate from his classmates during class so he can *think* clearly in class; and that he can *feel* connected to his friends by inviting them over to *communicate* together after school. This parenting may impact John’s thoughts and feelings during school. If John’s parents do not talk with him about this topic, John may experience different thoughts and feelings during school hours. When kept to one-self, thoughts and emotions are foundational to an *internal sense of self* as one functions in the larger society; and, when acted upon, thoughts and feelings can become verbal communication (e.g. speech) and non-verbal communication (e.g. body language, facial expressions) that form an *external sense of* self that is visible to the society. The (internal) experience of and (external) communication of thoughts, feelings and actions form the foundation of all systems of psychotherapy (Prochaska and Norcross, 2018). This view can be particularly helpful as researchers begin to investigate how smart technology impacts developmental changes to the self within the society beginning in youth.

#### Third: Concurrently ask questions at and between levels

Perhaps, a clinical researcher is interested in investigating protective mental health factors in the iGeneration; and they hypothesize that lower rates of loneliness, anxiety, and depression will be seen in subjects that do not respond to text messages immediately. The researcher might investigate further by using the philosophy to develop questions for the research subjects: “(1) Do you use conscious decision-making (self-precision) to prevent yourself from habitually responding to your phone when a text appears (self-variation)? (2) How fast do other’s in your social group typically respond to texts (society-variation)? (3) What changes in thoughts and feelings are experienced (internal self-society connection) after you communicate via text (external self-society connection)?” Perhaps this researcher also wants to investigate how those who are addicted to the technology perceive non-responders. The clinical researcher might again apply the philosophy: “(1) How fast do other’s in your social group typically respond to your texts (society-variation)? (2) Do you experience changes in thought and feeling (internal self-society connection) when others do not respond to you within an hour (society-variation)? (3) How do you communicate those thoughts and feelings (external self-society connection) with others when they do not respond for a prolonged period of time (society-variation)?” Future research might use this method to gather and organize levels of information on mental health factors across different self- and societal-conditions.

The processes that form our mental health form a functional connection between self and society. If mental health is a reflection of the self–society connection, what might be a reflection of the self–cell connection? Physiological health evidences a functional connection between our sense of self and our cells. For example, aerobic exercise is a health behavior that stimulates changes to variations in breathing and movement. By engaging in this behavior, the biological cells of the body are also stimulated via various physiological processes. Breathing will stimulate cellular functioning via the cardiovascular and respiratory systems; and movement will stimulate cellular functioning via the cardiovascular, musculoskeletal, and central nervous systems. While all physiological systems are working in collaboration in the body, certain changes to behavioral and biological functioning will stimulate certain physiological systems. By viewing health through this lens, between-level observations join the philosophy: biological functions emerge at the level of the cell; physiological functioning emerges as the cell–self connection; behavioral functions emerge at the level of the self; psychological/mental functioning emerges as the self–society connection; and social functions emerge at the level of the society. Future papers will explore maintainable-ease of functioning *at* and *between* levels.

### Future directions: new images of healthcare integration and new perspectives of healthcare innovation

**Fig. 4:**
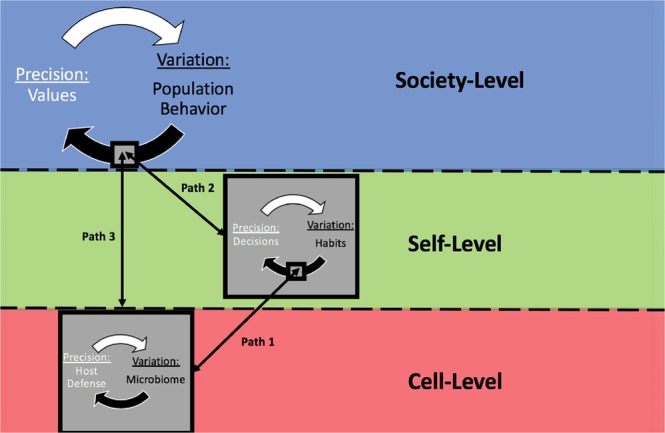
Integration of cell, self and society. Population health is generated along common paths that integrate the levels. The biological functioing of cells impacts fluctuations of habits/habitual functioning; and vice versa. The behavioral functioning of each self impacts fluctuations in population behavior; and vice versa. The biological functioning of cells also can impact fluctations in population behavior; and vice versa.

By considering this integrative philosophy, one can define health based upon a tangible connectedness, rather than separateness, of cells, selves, and societies. We provide Image 4 as a way to visualize the common paths to the health of healthy publics. When researchers observe that a host defense system is changing cellular functions following an infection, they may also expect these changes to have an impact [along Path 1] on expressions of habitual or physiological functions (e.g. immune function can stimulate the sensation of “achiness” or “pain” altering one’s physical movement, breath rate, hydration, and hunger) (Kelley, 2003; Johnson et al., 1992; Danzer, 2009). When researchers observe an individual deciding to engage in health behavior change following an addiction, they may also expect these changes to have an impact [along Path 2] on the group-behavior of their family system or social systems. When researchers observe changes to society’s values following a newly detected problem (e.g. laws ban Cigarette Advertising in broadcasting media; public health standards mandate certain vaccines before attending school), they may also expect that these changes can have an impact on behavioral functions of individuals [along Path 2] and biological functions of cells/organs [along Path 3]. These levels are continually integrating along these common paths to the health of healthy publics (Fig. 4).

When attending to this connectedness new, important questions can have new answers. *What function does modern technology serve in population health and healthcare?* If technology algorithms *prioritize* variations in population behaviors, then they fulfill a role as society-level precision. When modern technologies like machine learning (ML) technology and Computer Tailored Interventions (CTI) prioritize patterns of population behavior, we can see profound impacts on social change in a society. Although one might argue that technologies can be used by individual-level functions, *the algorithms* that are currently deployed and updated on devices interface with big-data gathered on population behaviors (Manogaran and Lopez, 2017; Dinov, 2016; Mullainathan and Spiess, 2017; Cheng et al., 2017).

In this paper, we identified that precision can be functional or dysfunctional. Similarly, technologies can support or prevent healthy population behavior. Some technologies prioritize health behavior in populations by tracking physical activity and providing feedback on activity progress; while others prevent healthy behavior by prioritizing sedentary behavior through video-gaming. Some social media technologies facilitate social communication with distant friends and relatives that supports wellbeing; while others facilitate conflictual communication that diminishes wellbeing. Given that modern technology can support or hinder health, we believe it is important that healthcare can prioritize technological innovations that value health in cells, selves, and societies. To do this, technology innovators might seek to value a higher order construct (e.g. maintainable-ease of functioning) in their algorithms.

Medical technology is currently used to titrate the doses of vaccines so that maintainable-ease of biological functioning (i.e. inoculation) is made available to the general population. When biological exposures are not properly titrated, infections can become active in the population and health is no longer valued at the level of the cell. Similarly, when behavioral and social exposures are not tailored to the needs of individuals and groups, populations can become resistant to healthy change, and health is no longer valued at the level of the self and the society. Behavior change researchers Prochaska and Prochaska (2016) report that when individuals and populations are not ready for a change, interventions that force individuals or populations to take action can increase resistance and prolong dysfunction. By tailoring (or what they term “staging”) behavioral and social level interventions, Computer Tailored Interventions upon behavioral and social functioning are made possible (Prochaska et al., 2001; Velicer et al., 2000, Prochaska and Prochaska, 2016). Despite these advances, there remains a need for technological advances that can make maintainable-ease of behavioral and social functioning available to the general population.

Future healthcare interventions could benefit from ML algorithms that tailor behavioral and social exposures to enhance precision-and-variation. Research already demonstrates that tailoring interventions for biological precision (Albert-Vega et al., 2018) and biological variation (Brodin et al., 2015) can impact long-term biological functioning. Future innovations might seek to use technology to tailor behavioral *and* social interventions to generate maintainable-ease of functioning. Through the functional language used in this paper we hope readers are inspired to present new questions, new comments, and new perspectives about needed healthcare innovations.
